# Study on the relationship between genetic polymorphism of reductive folic acid carrier and the risk of neural tube defects

**DOI:** 10.1007/s00381-022-05805-z

**Published:** 2022-12-20

**Authors:** Xusen Yang, Guofeng Fan, Zengliang Wang, Shaoshan Li, Hu Qin, Yun Wang, Xiaohu Ma, Wenyu Ji, Yongxin Wang

**Affiliations:** 1 Department of Neurosurgery, Xinjiang Medical University Affiliated First Hospital, Urumqi, 830054 China; 2 Department of Neurosurgery, Hami Hongxing Hospital, Hami, 839099 China

**Keywords:** Folic acid, Gene polymorphism, Neural tube defects, Mononucleotide, Association study

## Abstract

**Background:**

To investigate the association of folate metabolism gene polymorphism with neural tube defects (NTDs) in Chinese population.

**Methods:**

The subjects were divided into two groups, 495 children with NTDs (NTD group) and 255 healthy children (control group).

**Results:**

The levels of folic acid, s-adenosine methionine (SAM), and Sam/s-adenosine homocysteine (SAH) in NTD group were lower than those in control group. There were significant differences in hey, SAH, and Sam levels between two groups, but there was no significant difference in folic acid content. High fever in early pregnancy, taking antiepileptic drugs, father’s exposure to organic solvents, folic acid deficiency, and mother’s diabetes were the important risk factors in NTDs. MTHFR 677C > T gene was a risk factor for NTD in children, while 1298A > C gene was a protective factor.

**Conclusion:**

Folic acid metabolism markers were different in NTD children and their mothers, and the overall trend showed that folate, SAM, and SAM/SAH levels were decreased, while Hcy and SAH levels were increased; MTHFR 677C > T gene of SNPs was a risk factor for the occurrence of NTDs, and MTHFR 1298A > C gene was a protective factor, and the environmental risk factor had a synergistic effect on occurrence of NTDs.

## Introduction

Neural tube defects (NTDs) are the most common and severe group of birth defects in humans, with a worldwide incidence of about 0.5–2.0% [1]. As a country with a large population, China is also a country with a high incidence of NTDs. About 80,000 to 100,000 children with NTDs are born every year, especially in rural areas. NTDs are one of the important causes of fetal and infant death and long-term disability in adults, which brings great mental pressure and economic burden to the families and society, and have become a serious public health problem [2, 3]. The closure of neural tube is a continuous and precisely regulated biological event, which is influenced by both genetic and environmental factors. NTDs are regarded as a disease caused by environmental teratological factors affecting the development of nervous system on the basis of multi-gene inheritance [4]. Previous studies have shown that the occurrence of this disease is characterized by family aggregation, and family studies have shown that the risk of first-degree relatives of patients may increase by 40 times. Pregnant women with a history of NTD production will also significantly increase the probability of occurrence of NTDs in their offspring [5]. On the basis of previous studies, NTD cases were further collected, and the sample size was expanded in this study, aiming to explore the association and function of folate metabolism gene polymorphism and NTDs in Chinese population.

## Materials and methods

### General materials

The research projects were carried out with the approval of the Ethics Committee (No: 20160218–42). The subjects were 495 children with NTDs (NTD group) and 255 healthy children (control group); meanwhile, single nucleotide polymorphisms (SNPs) information from the parents of children with NTDs was obtained for statistical analysis, and the association between NTDs and genetic background, folic acid intervention, and exposure history of risk factors was investigated. In the control group, NTDs, congenital heart disease, mental retardation, and other birth defects or major diseases were excluded, including 146 males and 109 females, with an average age of 2.13 ± 1.02 years; the majority of severe abnormalities in the NTD group were detected by routine ultrasound during pregnancy (12–16 weeks), and NTDs were confirmed after birth in 283 males and 212 females, with an average age of 2.23 ± 1.14 years. There was no significant difference between the two groups (*P* > 0.05); there was no significant difference in parental age composition between NTD group and control group (*P* > 0.05); details are shown in Table 1. On the basis of informed consent of all subjects, general information was examined by specialists, including sociodemographic characteristics, folic acid intervention, and exposure to risk factors like smoking, alcohol consumption, high fever in early pregnancy, exposure to organic solvents, and use of antiepileptic drugs.

**Table 1:** Comparison of parental age composition between NTD group and control group

Group		*n*	Age (years)				*P*
			< 20	20–24	25–29	≥ 30	
Mother	NTD group	495	24 (4.85)	154 (31.11)	195 (39.39)	122 (24.65)	0.847
	Control group	255	13 (5.10)	75 (29.41)	101 (39.61)	66 (25.88)	
Father	NTD group	495	15 (3.03)	128 (25.86)	212 (42.83)	140 (28.28)	0.892
	Control group	255	8 (3.14)	64 (25.10)	105 (41.18)	78 (30.59)	

### Research methods

The association between NTDs and genetic background, folic acid intervention, and risk factor exposure was investigated on the basis of clinical data; specific biomarkers of folic acid and its metabolites homocysteine (Hcy), s-adenosine homocysteine (SAH), and s-adenosine methionine (SAM) were detected in each group, to explore the relationship between multiple SNPs and NTDs in offspring and to evaluate the relative risk of SNPs and NTPs; logistic regression analysis was used to elucidate the interaction of SNP gene, SNP, and environmental factors and to evaluate and predict the imaging effect of point mutation on protein function.

### DNA extraction

**Fig. 1:**
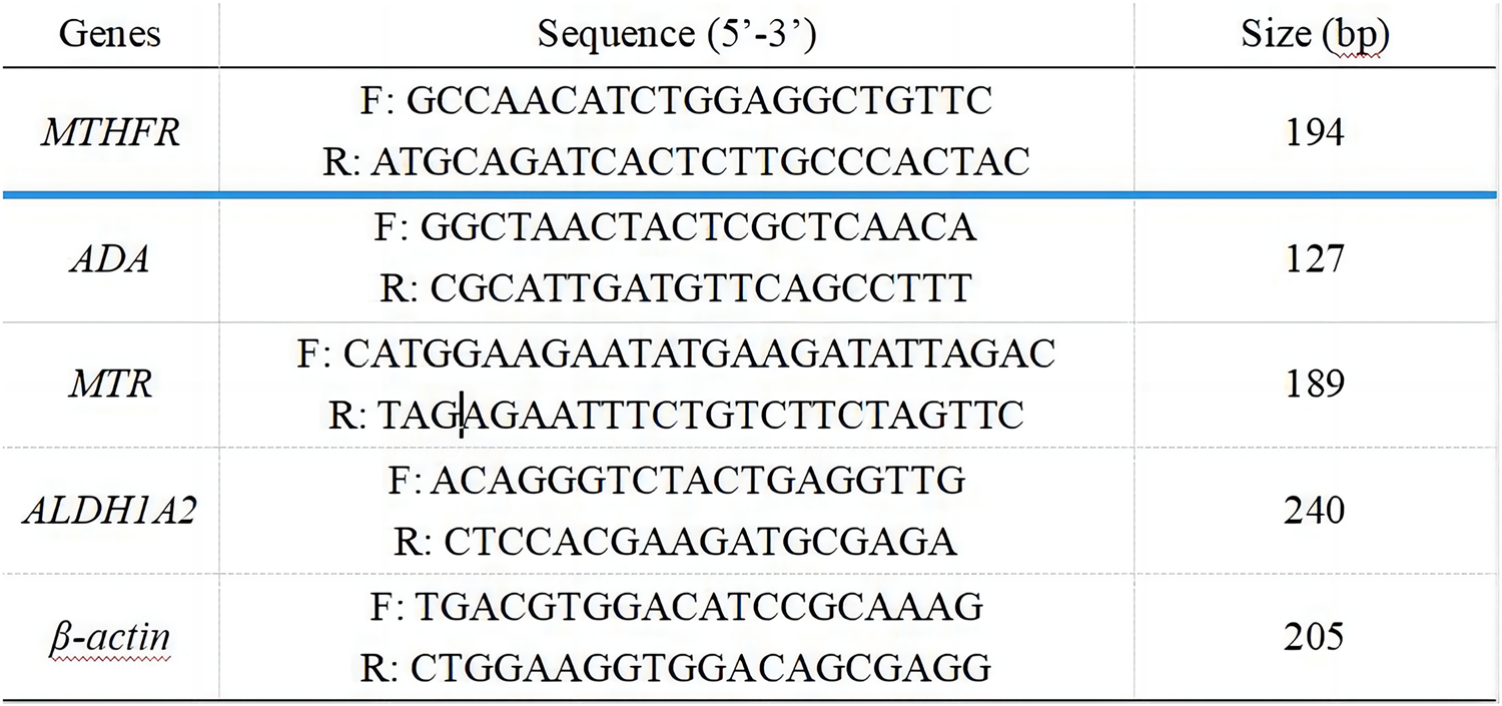
Primer and product design

Five milliliters of peripheral venous blood of subject was collected routinely, and the supernatant was taken after centrifugation with EDTA anticoagulation. Genomic DNA was extracted from white blood cells by QIAamp DNA Blood Maxi Kit (QIAGEN). This method was simple, time-saving, low cell consumption (200 μL WBC layer), and high yield. DNA concentration (50–100 ng/μL) and purity (uv 260OD and 280OD ratio between 1.8 and 2.0), all of which met the requirements of research. Folate metabolism-related genes were screened, and primers were designed to amplify DNA fragments containing SNPs. Primer and product design are as Fig. 1.

### SNP selection [5]

SNP selection is intended to proceed simultaneously from two aspects: functional selection and label selection. Firstly, potential functional loci of relevant genes were screened according to PubMed database, including promoter region SNPs, coding region SNPs causing missense mutations, and 3′UTR region SNPs. The selected loci were MAF ≥ 0.05 in the corresponding population. Secondly, according to HapMap database, Haploview software was applied to screen tag SNPs in the block where the reported susceptibility loci (rs1801133, rs452159, rs10925260, rs7169289) were located, and the SNPs that met the corresponding conditions were selected. The reported susceptibility loci MTHFR rs1801131 and MTR rs1805087 were forcibly included, and then the potential functions of SNPs in corresponding regions were preliminarily predicted according to SNPinfo website, and the linkage imbalance was combined to screen tag SNPs for association study. See Table 2 for SNP selection in this study.

**Table 2:** SNP selection

Source	Gene	rs number	The change of base	MAF	Location
Functional selection	*MTHFR*	rs1537514	G/C	0.1	UTR-3
		rs1537516	G/A	0.095	UTR-3
		rs1801131	T/G	0.198	missense
		rs1801133	G/A	0.488	missense
		rs2274976	T/C	0.093	missense
		rs3737967	G/A	0.102	missense
		rs4846048	A/G	0.105	UTR-3
		rs4846049	G/T	0.2	UTR-3
		rs13306561	G/A	0.093	nearGene-5
		rs17367504	A/G	0.093	nearGene-5
		rs3753582	C/A	0.1	nearGene-5
		rs3753584	T/C	0.093	nearGene-5
		rs7553194	A/G	0.095	UTR-5
	*ADA*	rs244076	T/C	0.151	cds-synon
		rs427483	C/G	0.222	intron
	*MTR*	rs1050993	A/G	0.209	UTR-3
		rs1050996	C/G	0.227	UTR-3
		rs10925263	C/T	0.256	UTR-3
		rs1804742	C/T	0.083	UTR-3
		rs1805087	A/G	0.07	missense
		rs2853522	A/C	0.465	UTR-3
		rs2853523	A/C	0.214	UTR-3
		rs3768159	C/T	0.209	UTR-3
		rs3768160	C/T	0.209	UTR-3
	*ALDH1A2*	rs1061278	T/C	0.314	UTR-3
		rs1063666	C/A	0.314	UTR-3
		rs3204689	G/C	0.178	UTR-3
		rs3204690	T/G	0.314	UTR-3
		rs4646626	T/C	0.314	missense
		rs4646642	G/A	0.298	UTR-3
		rs4646643	G/A	0.128	UTR-3
		rs4646644	C/A	0.128	UTR-3
		rs9325	A/T	0.186	UTR-3
Label selection	*MTHFR*	rs4846049	G/T	0.2	UTR-3

### Statistical processing

The relevant data of the study were statistically processed by SPSS 20.0 software, the measurement data (x ± s) were tested by independent sample t, and the counting data (%) were tested by Chi-square x^2^. Setting: *P* < 0.05 was considered statistically significant and vice versa.

Logistic regression analysis was used to clarify the interaction between SNPs (gene–gene) and SNPs with environmental factors (gene-environment), to explore the ways and intensity of interaction and to compare the commonalities and specificity in different types of neural tube defects; through in vitro molecular biology studies, functional interpretation of SNP sites with significant associations in specific regions was carried out, including the detection of the transcriptional regulation effect of SNPs on the host gene by using the dual-luciferase reporting system and the detection of the expression of genes with different SNPs sites by RT-PCR.

## Results

### Comparison of levels of folate metabolism markers between NTD group and control group

The levels of Hcy and SAH in the mothers of children in the NTD group were significantly higher than those in the control group, while the levels of folic acid, SAM, and SAM/SAH were lower than those in the control group. Besides Hcy, there were statistically significant differences in the levels of other markers between the groups (*P* < 0.05); there was a statistically significant difference in Hcy level between NTD group and control group (*P* < 0.05). The levels of Hey, SAH, and SAM in children of NTD group and control group were statistically significant (*P* < 0.05), while the levels of folic acid were not statistically significant (*P* > 0.05). Details are shown in Table 3.

**Table 3:** Comparison of the levels of folate metabolism markers between NTD group and control group

Markers		NTD group (*n* = 495)	Control group (*n* = 255)	*P*
Mother	Folic acid (nmol/L)	7.5 ± 1.6	9.4 ± 1.5	< 0.001
	Hcy (μmol/L)	12.4 ± 3.3	11.2 ± 2.2	0.059
	SAM (nmol/L)	49.7 ± 2.9	53.1 ± 3.2	< 0.001
	SAH (nmol/L)	12.9 ± 1.6	10.0 ± 2.1	< 0.001
	SAM/SAH	3.8 ± 0.6	5.5 ± 1.3	< 0.001
Father	Folic acid (nmol/L)	7.8 ± 2.2	8.2 ± 2.1	0.472
	Hcy (μmol/L)	13.2 ± 2.4	12.1 ± 2.6	0.033
	SAM (nmol/L)	51.4 ± 2.8	52.1 ± 2.7	0.147
	SAH (nmol/L)	11.5 ± 1.8	11.2 ± 1.7	0.215
	SAM/SAH	4.6 ± 0.8	4.7 ± 0.5	0.063
Children patient/children	Folic acid (nmol/L)	8.7 ± 1.5	9.5 ± 1.6	0.076
	Hcy (μmol/L)	9.6 ± 1.7	8.4 ± 1.6	0.011
	SAM (nmol/L)	51.1 ± 2.5	52.2 ± 2.6	0.025
	SAH (nmol/L)	10.5 ± 1.8	9.8 ± 1.7	0.014
	SAM/SAH	4.9 ± 0.6	5.4 ± 0.9	< 0.001

### Investigation of risk factors related to NTDs

High fever in early pregnancy, use of antiepileptic drugs, paternal exposure to organic solvents, lack of folic acid, and maternal diabetes were important risk factors for NTDs (*P* < 0.05). Details are shown in Table 4.

**Table 4:** Investigation of risk factors associated with NTDs

Risk factors	OR	95% CI	*P*
Smoking	4.8	0.725–9.328	0.738
Alcohol consumption	3.3	0.891–3.954	0.069
High fever in early pregnancy	8.9	1.253–12.627	< 0.001
Use of antiepileptic drugs	10.4	2.530–18.892	< 0.001
Paternal exposure to organic solvents	5.7	2.248–9.336	0.012
Lack of folic acid	12.3	6.085–34.326	< 0.001
Maternal diabetes	6.2	1.089–8.874	0.007

### Screening SNP loci information

**Fig. 2:**
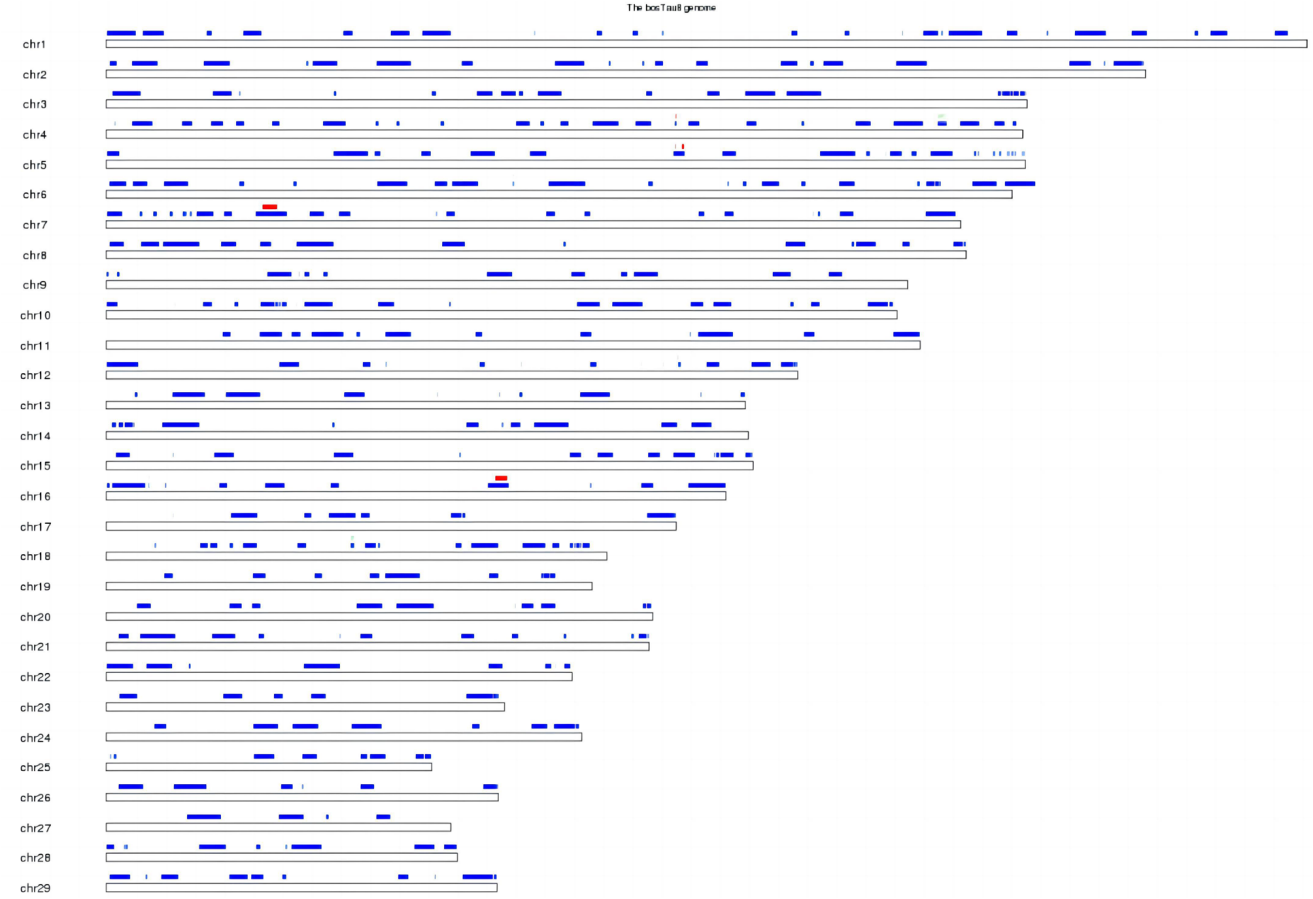
Distribution of CNV chromosome

In this study, 33 functional SNP loci were selected, 1 tag locus was selected, and 29 chromosomes were detected. The distribution of CNV chromosome is shown in Fig. 2. The general information of loci is shown in Table 5.

**Table 5:** Genotype distribution and Hardy–Weinberg equilibrium test

Gene	Nucleotide change	Children patient/children		Mother		Father	
		x^2^	*P*	x^2^	*P*	x^2^	*P*
MTHFR	c.677C > T	− 5.24	< 0.05	− 6.61	< 0.001	− 4.28	0.013
	c.1298A > C	4.36	< 0.001	5.03	< 0.001	3.72	< 0.001

### Genotype distribution and Hardy–Weinberg equilibrium test

MTHFR 677C > T gene and MTHFR 1298A > C gene of SNPs were associated with pregnant women with neural tube defects in children (*P* < 0.05), and 677C > T gene was a risk factor, while 1298A > C gene was a protective factor. Environmental risk factors have a synergistic effect on the occurrence of NTDs. Details are shown in Table 6 and Table 7.

**Table 6:** Gene-environmental risk factor association

Gene	Nucleotide change	Use of antiepileptic drugs		Lack of folic acid		Maternal diabetes	
		x^2^	*P*	x^2^	*P*	x^2^	*P*
MTHFR	c.677C > T	− 7.38	< 0.001	− 9.32	< 0.001	− 7.65	< 0.001
	c.1298A > C	12.34	< 0.001	10.88	< 0.001	9.74	< 0.001

**Table 7:** Gene-environmental risk factor association

Gene	Nucleotide change	Use of antiepileptic drugs		Lack of folic acid		Maternal diabetes	
		x^2^	*P*	x^2^	*P*	x^2^	*P*
MTHFR	c.677C > T	− 7.38	< 0.001	− 9.32	< 0.001	− 7.65	< 0.001
	c.1298A > C	12.34	< 0.001	10.88	< 0.001	9.74	< 0.001

## Discussion

The formation of neural embryo is the earliest and most critical period in the development of human embryo, and the neural tube begins to form; from the third week of pregnancy, the nerve plate bulges, nerve folds appear close together and fuse, and forms such as the closure of neural fora begin to develop. If there are risk factors affecting the development of neural tube, the closure may be incomplete, i.e., NTDs [6]. NTDs are a severe congenital defect of the central nervous system, with an incidence of about 1–2%. The incidence varies from people to people. Studies have shown that folic acid supplementation during pregnancy can reduce the incidence of NTDs by 60–70%, which has also been confirmed by clinical trials [7]. Folic acid is a carbon unit donor found in many plant and animal tissues, a source of substrate for DNA synthesis, and provides nutrients for neural tube development during the development of embryo. Mammals are unable to synthesize folic acid from the beginning and rely on dietary intake. The proximal intestinal villi absorb the mono-glutamate of folate through RFC for utilization. Folate enters the blood in the form of 5-methyltetrahydrofolate and enters cells through folate receptor and RFC. Folate-carbon metabolism is an interconnect reaction network. A large number of clinical studies have suggested that if folic acid supply is insufficient or metabolism is abnormal during pregnancy, it may affect the development of embryonic neural tube and lead to incomplete closure of neural tube. Studies related to neural tube development model have suggested that folic acid level can affect the differentiation process of human embryonic stem cells into neural stem cells, and folate supplementation can reverse this differentiation, which may be one of the pathogenesis of NTDs and a clinical approach to prevent NTDs [8]. Folic acid deficiency can lead to fetal neural tube malformation, congenital cleft lip and palate, and hyperhomocysteinemia, and is also closely related to the occurrence and development of some birth defects. Therefore, association studies about folic acid metabolism and birth defects have attracted increasing attention [9]. Folate metabolism involves a variety of enzymes; the key enzymes mainly include 5,10-methylene tetrahydrofolate reductase (MTHFR), methionine synthase (MTR), methionine synthase reductase (MTRR), and cysteine synthase (CBS), among which MTHFR and MTR are two key enzymes involved in folate activation and methyl donor generation [10]. The genes of the enzymes have single nucleotide polymorphisms in the population, which can change the enzyme activity and cause abnormal folate synthesis and metabolism in vivo, thus leading to DNA synthesis disorders and DNA methylation abnormalities, which may lead to birth defects [11]. In this study, children in the NTD group and those in the control group and their parents were investigated. The data showed that the levels of Hcy and SAH in the NTD group were significantly higher than those in the control group, while the levels of folate, SAM, and SAM/SAH were lower than those in the control group; the difference of Hcy level between NTD group and control group was statistically significant. There were significant differences in the levels of Hey, SAH, and SAM between NTD group and control group, but there was no significant difference in folic acid levels. These results have suggested that the level of folic acid and its metabolites is related to the occurrence of NTDs, and monitoring the changes of these indicators during pregnancy can provide a basis for early clinical diagnosis of NTDs. In addition, previous studies have suggested that high fever in early pregnancy, use of antiepileptic drugs, and paternal exposure to organic solvents may be associated risk factors for NTDs. In this study, two groups of risk factors were investigated, and the results showed that high fever in early pregnancy, use of antiepileptic drugs, paternal exposure to organic solvents, lack of folic acid, and maternal diabetes were all important risk factors for the occurrence of NTDs. In view of this, NTDs can be predicted and prevented by understanding the high risk factors of both parents during routine prenatal examination.

Domestic and foreign studies have shown that folate metabolism has an important relationship with the occurrence of NTDs. Considering environmental factors, folate metabolism-related genes are the most interesting candidate genes in the occurrence of NTDs [12]. The genes which have closer association with NTDs, like MTHFR, ALDH1A2, ADA, and MTR, all belong to folic acid in the metabolic pathway enzyme gene, MFTC(SLC25A32) belongs to transport protein gene, ARID1A coding is a transcription factor, PRICKLE belongs to genes related to embryo development, and FUT2 is associated with the immune processing of post pathogen infection [13]. Existing studies have shown that changes in BHMT gene 716G > A can trigger the development of Arg239Gln, changes in MTHFD1 gene 1958G > A can trigger Arg635Gln, changes in MTR gene 2756A > G can trigger Asp919Gly, and so on [14]. Folic acid metabolism and methyl group supply constitute a very complex biomolecular metabolic network, which is the result of multi-factor and multi-link cooperation. It is difficult to obtain ideal results by analyzing a single marker or gene locus alone. SNPs are genomic polymorphisms in DNA sequences caused by single nucleotide mutations, which are most common in human heritable variants. It can not only be used as a genetic marker to locate disease genes through linkage analysis, but also directly lead to the occurrence of diseases. Therefore, in clinical practice, SNPs are regarded as an important part of early disease risk assessment, diagnosis, and prevention [15]. Based on existing studies, this study further expanded the sample size of NTD cases. The association analysis results of NTDs and SNPs showed that the MTHFR 677C > T gene of SNPs and MTHFR 1298A > C gene were associated with pregnant women with neural tube defects in children, and the 677C > T gene was a risk factor, while the 1298A > C gene was a protective factor; environmental risk factors have a synergistic effect on the occurrence of NTDs. As a key enzyme of folate metabolism, MTHFR play an important role in the process of DNA methylation and DNA synthesis and repair. In the Chinese population, the changes of 677C > T and 1298A > C of the MTHFR gene of the fetus and its parents were both related to the occurrence of NTDs, and the changes of 677C > T significantly increased the risk of the occurrence of NTDs, which also confirmed that the rs1801133 site was associated with the susceptibility of NTDs. Furthermore, changes in MTHFR 677C > T gene and MTHFR 1298A > C gene combined with environmental risk factors can further increase the risk of NTDs in embryos.

## Conclusion

In conclusion, the levels of folic acid metabolism markers of NTD children and their mothers were different, and the overall trend showed that the levels of folic acid, SAM, and SAM/SAH decreased, while the levels of Hcy and SAH increased. The MTHFR 677C > T gene of SNPs is a risk factor for the occurrence of NTDs, and the MTHFR 1298A > C gene is a protective factor, and the environmental risk factor has a synergistic effect on the occurrence of NTDs.

## Data Availability

The datasets generated and/or analyzed during the current study are available in the China National Science Foundation Network Information System repository, [https://isisn.nsfc.gov.cn/].

## Abbreviations

NTDs: Neural tube defects
SAM: S-adenosine methionine
SAH: Sam/s-adenosine homocysteine
MTHFR: Methylenetetrahydrofolate reductase
SNPs: Single nuclear tide polymorphisms
Hcy: Homocysteine
EDTA: Ethylenediaminetetraacetic acid
MTR: 5-Methyltetrahydrofolate-homocysteine methyltransferase
DNA: Deoxyribonucleic acid
UTR: Untranslated regions
MAF: Minor allele frequency
CNV: Copy number variation
ALDH1A2: Aldehyde dehydrogenase 1 family member A2
ADA: Adenosine deaminase
MFTC(SLC25A32): Solute carrier family 25 member 32
ARID1A: AT-rich interaction domain 1A
PRICKLE: Prickle planar cell polarity protein
FUT2: Fucosyltransferase 2
BHMT: Betaine–homocysteine S-methyltransferase
MTHFD1: Methylenetetrahydrofolate dehydrogenase cyclohydrolase and formyltetrahydrofolate synthetase
